# The impact of residential environment on older people’s capabilities to live independently: a survey in Beijing

**DOI:** 10.1186/s12889-024-18262-x

**Published:** 2024-03-18

**Authors:** Mengyuan Chen, Gideon Bolt, Pieter Hooimeijer

**Affiliations:** Department of Human Geography and Spatial Planning, Faculty of Geosciences, Utrecht University, Princetonlaan 8a, 3584CB Utrecht, The Netherlands

**Keywords:** Sen's capability approach, Community-based intervention, Residential environment, Older people

## Abstract

**Background:**

Studies have shown how environmental factors influence older people's health and functional limitations, which are crucial for achieving healthy aging. However, such a healthy aging model has been criticized for defining health as an absence of disease, because chronic conditions cannot be reversed through medical treatments. In response to such critiques, this study refers to Huber's positive health definition, arguing that health should not be defined as the absence of disease but as the ability to adapt and self-manage in the face of social, physical, and emotional challenges. There is a need to develop a community-based approach to healthy aging that considers how the residential environment enables older people to adapt and self-manage. Drawing on Sen's capability approach, this study proposes that such a community-based approach should provide a supportive environment to enable older people's capabilities to live independently.

**Methods:**

Using hierarchical multiple regression analysis of data from 650 older people (60 years and older) surveyed in Beijing, we unravel which features of the residential environment support older people' s capabilities to live independently and how these impacts differ depending on older people's frailty levels.

**Results:**

The results show that four environmental factors, namely perceived accessibility (B = 0.238, *p* < 0.001 for physical capability, B = 0.126, *p* < 0.001 for social capability, B = 0.195, *p* < 0.001 for psychological capability), pleasant surroundings (B = 0.079, *p* < 0.05 for physical capability, B = 0.065, *p* < 0.05 for social capability), meeting opportunities (B = 0.256, *p* < 0.001 for social capability, B = 0.188, *p* < 0,001 for psychological capability, and life convenience B = 0.089, *p* < 0.05 for physical capability, B = 0.153, *p* < 0.001 for psychological capability) positively affect older people's capabilities to live independently. These four environmental factors cause differences in older people's capabilities between different neighborhood types. Moderation analysis shows that meeting opportunities are more relevant for frail older people (B = 0.090, *p* < 0.001 for social capability, B = 0.086, *p* < 0.01 for psychological capability).

**Conclusions:**

This study contributes to the literature by emphasizing the role of supportive residential environments in enabling older people to live independently. Furthermore, we identify four environmental factors that support older people's capabilities. Results can be used to develop effective community-based environmental support to enable older people to live independently.

## Background

Environmental factors have been shown to influence the health of older people and their functional limitations, suggesting that environmental support is crucial for achieving healthy aging [1, 2]. Such a healthy aging model defines health as an absence of disease with a particular focus on conquering diseases and extending individual lives [3, 4]. However, by definition, chronic conditions cannot be reversed through medical treatments. The notion that older people with chronic conditions are ill underestimates the human capacity to maintain and restore one's integrity and independence [3, 4]. Therefore, this study refers to Huber's positive health definition [4], arguing that health should be defined as the ability to adapt and self-manage in the face of physical, social, and emotional challenges. According to Huber's health conceptualization, older people with chronic conditions can still maintain a healthy lifestyle [3, 4]. There is a need to develop a framework that integrates community-based support to enable older people to adapt and self-manage (or to live independently).

Sen's capability approach offers a framework that suggests community-based environmental support that enables older people to adapt and self-manage. In the literature, there is a recognition that beyond the home, features of the neighborhood environment can support older people [5–8]. According to Lawton's environmental-fit theory, adaptive functioning in the environment depends on the interaction between a person's environment that places demands on that individual (environmental press) and the individual's competencies [9]. In other words, older people's quality of life can be affected by the supportive residential environment. However, rather than seeking the adaptive functioning of older people, the fundamental insight of Sen's capability approach is that the policy focus should be on older people's capabilities [10–14]. In the capability approach, Sen defines capabilities by distinguishing them from functionings. Whereas functionings are "beings and doings" (actual achievement), capabilities refer to the opportunities of accessing valued functionings (opportunity to achieve). For example, if being well-nourished is a functioning, then being able to access sufficient food is a corresponding capability [11, 13]. The concept of capability emphasizes multiple dimensions of human development, such as being able to live a satisfying life, being able to have fun, and being able to associate with other people [13]. Therefore, the capability measures are different from the disability measures, it emphasizes the importance of 'being able to' live independently (physically, socially, and psychologically) despite having chronic conditions. In other words, even when older people become frail, they can lead lives they value. This multidimensional view of health is relevant because it argues that aging is not (only) defined by declining physical health. More importantly, the capability approach emphasizes the capabilities that older people have reflect a person's access to environmental resources [8, 10]. It is thus crucial to know how the residential environment enables older people's capabilities to live independently. Nevertheless, specific ways that environmental factors affect older people's capabilities to live independently are unclear. This is because various features characterize the residential environment, and the choice of the valued capabilities varies from person to person according to their valued capabilities [14, 15]. Such extensive diversity makes it difficult to compile a list of environmental factors [13, 15].

Notably, Sen's capability approach has the advantage of addressing the issue of human diversity [13]. In the capability approach, Sen addresses the concept of the conversion factor, acknowledging that different older people with different characteristics have different abilities to convert resources into capabilities [13, 15]. Frailty may be one of the personal conversion factors. Frailty is a geriatric condition that makes an individual susceptible to external stressors [16]. Studies have shown that older people's needs for an environment are highly likely to vary depending on their frailty level [17], which implies that the level of frailty as a personal conversion factor influences the degree to which the residential environment affects older people's capabilities to live independently.

This study uses a survey of older people in Beijing, China. The population in China is aging at an unprecedented rate, posing concerns about the provision of care for older people. According to the Seventh National Population Census, there are 190.64 million older people (over 65) in China, accounting for 13.50% of the country's total population [18]. Older people are more likely to suffer from chronic diseases, and many of them require (health) care services such as housekeeping, nursing, and psychological counseling [19, 20]. Worse still, the declining capacity of family networks to provide care has led to an increasing number of older people living in institutional care facilities [19, 21]. As a result, there is pressure on the healthcare system [21]. In order to relieve the costs of healthcare, policymakers in Beijing prioritize community-based initiatives, arguing that older people should live independently in their homes through community-based support [22]. The Beijing municipal government announced that vulnerable older people should receive a wide range of community-based support. A large variety of programs are offered, including aging-friendly modifications for older people with physical disabilities, renovation of old dilapidated residences, and the creation of community centers for calligraphy, chess, and painting activities. Additionally, there are efforts to ensure access to facilities such as exercise facilities, bus stops (subways), pharmacies, and vegetable markets within walking distance [22–25]. Based on a survey of older people in Beijing, we used hierarchical multiple regression analysis to answer the following research questions:

Which features of the residential environment support older people's capabilities to live independently? How do these impacts differ depending on older people's frailty levels?

## Methods

### Conceptual framework

#### How frailty affects older people's capabilities

When people grow old, chronic conditions cause them to become frail, and several challenges may occur with frailty which may negatively affect older people's capabilities to live independently. First, frail older people are more likely to face physical impairments, such as tiredness and walking difficulties [26]. They may be unable to perform daily tasks and errands. Second, frail older people have an increased risk of loneliness and isolation because of a decline in social networks [26]. They may be unable to connect with others and participate in community activities and public affairs. Third, older people may suffer from depression and anxiety and find it difficult to cope with challenges [26]. Accordingly, we expected that a community-based environmental intervention should support older people's capabilities to live independently. Such capabilities can be divided into physical capabilities (occupational performance), social capabilities (social networks), and psychological capabilities (mental health). Physical capability is related to body-related occupational performance crucial to maintaining daily routines, which refers to an older person's ability to perform daily tasks (such as work, education, housework, family, and leisure activities), as well as life safety concerns when performing these tasks [27]. Social capability refers to older people’s ability to maintain social relationships, such an ability involves participating in community activities and public affairs as well as interacting with others [26, 28, 29]. Notably, a valued social capability for Chinese older people is the ability to play a supportive role in the family. Due to Confucian ideology that encourages family interaction and support, older people feel obligated to continue caring for younger generations even as they grow old [30]. Psychological capabilities refer to emotional coping strategies such as "stopping unpleasant feelings and thoughts" [31] and problem-based coping strategies [31] that will enable them to deal with challenges.

#### The role of a supportive residential environment

Supporting three dimensions of capabilities requires attention to the community rather than individual. Nevertheless, China has four types of neighborhoods with vastly different residential environments and demographic composition: traditional neighborhoods, former danwei compounds, commercial housing, and urban villages [32–34]. Traditional neighborhoods are the oldest residential areas in the inner city. In most of the houses, there is a communal kitchen and bathroom, a semi-closed corridor, and a central shared courtyard. The condition of courtyard facilities is deteriorating, making it impossible for older residents to meet their needs. Most of its residents are older people are laid-off workers [32–34]. Former danwei compounds were apartments built for employers during socialist times. In the former danwei compounds, most apartments range from 61 to 80 square meters, with one or two bedrooms. Each unit has a toilet and a closed kitchen area. The residents of this area were former employees of Danwei. The conditions of these neighborhoods are generally better, often with walkable pavements and greening spaces. Facilities such as pharmacies and vegetable markets were all integrated in close proximity [32–34]. Commercial housing consists of apartment buildings built by private developers. Apartment buildings generally have aesthetically pleasing interiors with large housing units measuring over 100 square meters. Property management services, beautiful landscaping, and elaborate security measures are all available in the neighborhood. Those living in Beijing's commercial housing were financially well-off [32–34]. In urban villages, we can find self-built apartments in multistory buildings divided into multiple one-room apartments available for rent. Migrants make up most of its population. Most of these neighborhoods offer basic necessities, but they are usually located outside of Beijing and not everything is easily accessible [32–34]. Since four types of neighborhoods vary greatly in the residential environment and demographic composition, we are interested in examining to what extent the residential environment explains the capability differences between different neighborhood types.

A further investigation is required to determine which features of the residential environment support older people's capabilities to live independently. We identify four environmental factors (perceived accessibility, pleasant surroundings, meeting opportunities, and life convenience). First is perceived accessibility. According to Iwarsson and Stahl [35], perceived accessibility should include both human and environmental components. This is because environments impose greater demands on some people while others do not. Older people with better functional capacity are likely to rate the physical environment as accessible, even though the same environment may be considered inaccessible by someone using a wheelchair [35]. Therefore, in the European ENABLE-AGE project [36], the fit between person-environment components is incorporated under perceived accessibility objectives. It refers to the extent to which the physical environment of a home supports the autonomy of older people in their daily activities. Perceived accessibility includes features such as ease of approach to the home, circulation, and ease of use in the kitchen and bathroom that enable disabled older people to participate in physical activities [27, 35, 37]. Second is a pleasant environment, which is a comfortable and safe living environment within the neighborhood. Typically, older people's comfort and security concerns are affected by the status of aging-friendly design, crime, and traffic [38, 39]. A tidy, well-maintained place offers older people comfort, privacy, and security [39, 40]. Exposure to the natural environment also stimulates this aspect. Studies have shown that a visual connection with nature and activity in natural green areas contributes to various psychological and health benefits [28, 41]. Older people's comfort and security concerns are also related to the prevalence of criminal activities such as mugging, attacks, and stealing. These illegal acts harm older people in their daily lives [39]. Many neighborhoods, especially those with high densities, also suffer from traffic hazards. Because older people mostly walk within the community, crossing pedestrian and vehicular routes is dangerous due to personal injuries [39, 41]. Third is the meeting opportunities that enable older people to interact with one another. Meeting opportunities help buffer loneliness, stress, and alienation and allow older people to be physically active and socially connected [39, 41, 42]. It also enables people to develop coping strategies to live independently, including sharing information, informal monitoring, and care coordination [29]. Informal meeting spaces can provide meeting opportunities since this is where older people gather for formal and informal socialization and entertainment [29, 42]. Having helpful and trustworthy neighbors also stimulates this aspect [29, 42]. In addition, in China, residents' committees are crucial institutions for promoting neighborliness, harmony, and cohesiveness; they are responsible for connecting with government authorities and making recommendations about the needs and aspirations of residents [43]. Fourth is life convenience, which is the proximity to services and amenities that support the daily needs of older people. Studies have shown that urban facilities such as locations for physical exercise [44], medical facilities [39], public transportation, retail, and recreational services are crucial to improving the quality of life for older people both physically and mentally [39, 45]. We explore how four identified environmental factors enable older people's capabilities.

Depending on the characteristics of older people, the residential environment can have a different impact on their capabilities to live independently. An important concept in Sen's capability approach is conversion factors, which acknowledge the fact that older people have different abilities to convert resources into capabilities [13, 15]. Frailty may be one of the conversion factors. Studies have shown that although frail and non-frail people share common desires for capabilities such as independence, security, and belonging, their meanings differ. A frail older person may feel independent with the support of home help, while a non-frail older person may feel independent by being able to clean their house by themselves [17]. The question is, therefore, whether frailty influences the degree to which the residential environment affects older people's capabilities to live independently.

#### Individual characteristics

Apart from frailty, several individual characteristics may also influence older people's capabilities to live independently. First, research has shown that social networks and support from family and friends contribute to better health and well-being [7]. Second, socioeconomic factors, such as level of education and income, influence older people's quality of life. For example, more educated older people receive more generous pensions and have better access to healthcare facilities than their less educated counterparts [28, 45]. Third, hukou in China is a crucial institutional factor affecting older people's health and well-being. People with a non-local hukou (migrants) do not have the same access to welfare provisions as those with a local hukou (local people). Therefore, residents with local hukou are generally in a more socially and economically privileged position [45]. Fourth, literature has documented gender differences in resource ownership and control among older people [46]. We hypothesized that these individual characteristics affect older people's capabilities to live independently.

**Fig. 1:**
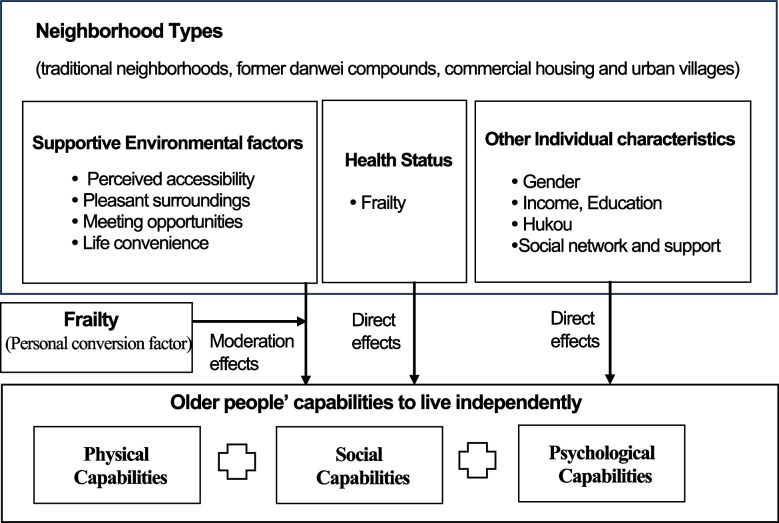
Theoretical framework

Figure 1 presents the conceptual framework to test three hypotheses: Hypothesis 1. Differences between four neighborhood types (traditional neighborhoods, former danwei compounds, commercial housing, and urban villages) in older people's physical, social, and psychological capabilities can be attributed to the inequalities in the residential environment. Hypothesis 2. Four environmental factors (perceived accessibility, pleasant surroundings, meeting opportunities, and life convenience) may positively affect older people's physical, social, and psychological capabilities. Hypothesis 3. Older people's frailty level may moderate the impact of the four environmental factors (perceived accessibility, pleasant surroundings, meeting opportunities, and life convenience) on older people's physical, social, and psychological capabilities.

### Data collection

Data for this study were derived from a questionnaire survey collected from April 2021 to July 2021 titled "Community-based Intervention for older people in China." Data should be collected from older people to test the hypotheses of the conceptual framework. The first part of the questionnaire was about the general information of respondents, such as age, gender, education, hukou status, income, marital status, and instrumental support older people received from their adult children (a five-point scale ranging from 1 to 5), and social networks (measured by an abbreviated version of Lubben's Social Network Scale that has six six-point scales ranging from 1 to 6) [45, 47]. We also included the cardiovascular health study (CHS) index to measure frailty. The CHS index measures frailty based on older people's biophysical syndrome and has a solid foundation in biological theory [16]. Weight loss, low physical activity, exhaustion, slowness, and weakness were the five indices of CHS. Using these five items, we formed a combination score.

The second part of the questionnaire developed a new measurement to assess the residential environment and capabilities (See Table 1 for more specific questions). The structure of the scale was confirmed based on a pilot survey of 220 respondents in the autumn of 2020. For environmental factors, KMO (0.87) and Bartlett's Test (χ2 = 2241.36, df = 136, p < 0.001) indicated that the scale was acceptable for factor analysis, and exploratory factor analysis (EFA) indicated a four-factor data structure. For capabilities, KMO (0.89) and Bartlett's Test (χ2 = 1411.74, df = 66, *p* < 0.001) indicated that the scale was acceptable for factor analysis, and exploratory factor analysis (EFA) indicated a three-factor data structure.

**Table 1:** Measurement items

	**Actual Survey questions**^**a**^
**Capability**	With regard to the experience of aging in place, to what extent do you agree with the following expressions?
Physical capability (Phy)	(Phy1) I am able to carry out daily tasks and errands (e.g., work, study, housework, family, or leisure activities)
	(Phy2) I am able to take care of myself
	(Phy3) I am able to meet the daily demands of life
	(Phy4) am able to get out and about with no security concerns
Social capability (Soc)	(Soc1) I am able to find some people to communicate with
	(Soc2) I am able to be socially engaged in community life
	(Soc3) I am able to keep up with current affairs in the public domain
	(Soc4) I am able to act as a supportive role in the family
Psychological capability (Psy)	(Psy1) I am able to take enjoyment from life
	(Psy2) I am able to maintain a cheerful outlook
	(Psy3) I am able to do things that I value
	(Psy4) I am able to cope with problems that occur with aging
**Environmental factors**	To what extent would you agree with the following descriptions of residential environment attributes?
Perceived accessibility (PA)	(PA1) The home is easily accessible from the neighborhood
	(PA2) Activities within the home are easy and barrier-free
	(PA3) Reaching above and below cabinets is easy
	(PA4) Have a well-furnished bathroom
	(PA5) Have a well-furnished kitchen
Pleasant surroundings (PS)	(PS1) There are sufficient greening services
	(PS2) There is no crossing of pedestrian and vehicular routes
	(PS3) Well-maintained neighborhood facilities and efficient cleaning are provided
	(PS4) There is a safe environment with no criminal activities
Meeting opportunities (MO)	(MO1) Have informal meeting spaces such as parks and squares
	(MO2) Have danwei or neighborhood committees hold activities
	(MO3) Have trustworthy neighbors that enable information sharing
	(MO4) Have helpful neighbors who help each other
	(LC1) Exercising facilities are nearby
Life convenience (LC)	(LC2) Bus stop and subway stations are nearby
	(LC3) Pharmacies and hospitals are nearby
	(LC4) Supermarkets and vegetable markets are nearby

^a^Note: Each item was rated on a 5-point Likert scale. 1-strongly disagree, 2- disagree, 3-neither agree nor disagree, 4- agree, 5- strongly agree

**Fig. 2:**
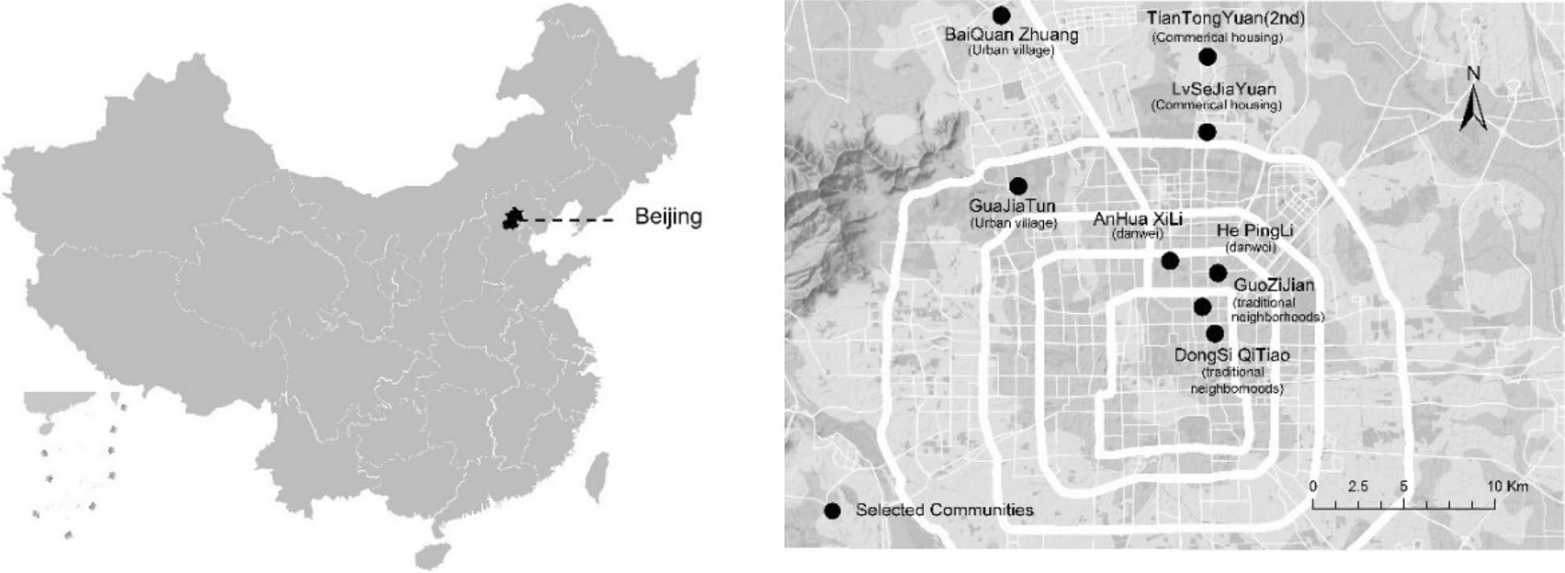
The geographical location of the studied area. (Left: Location of Beijing in China. Right: selected communities in Beijing)

This paper selected Beijing as the study area because Beijing is one of the most populated cities that age rapidly. Statistics from the 7th Census shows that 19.6% of residents in Beijing age 60 and older [18]. We used a stratified sampling method to ensure the sample approximates the older population living in different residential environments (See Fig. 2). Each of the four types of residence (traditional neighborhoods, former danwei compounds, commercial housing, urban villages) was represented by two communities and we sampled 100–200 older people from eight communities. The selection of the community is based on the administrative boundaries of the residents' committee (communities) in China (See Fig. 2).This study required respondents to be frail older people aged 60 and older. 1,283 respondents were approached, and 714 respondents completed the questionnaire.

### Data analysis procedures

**Fig. 3:**
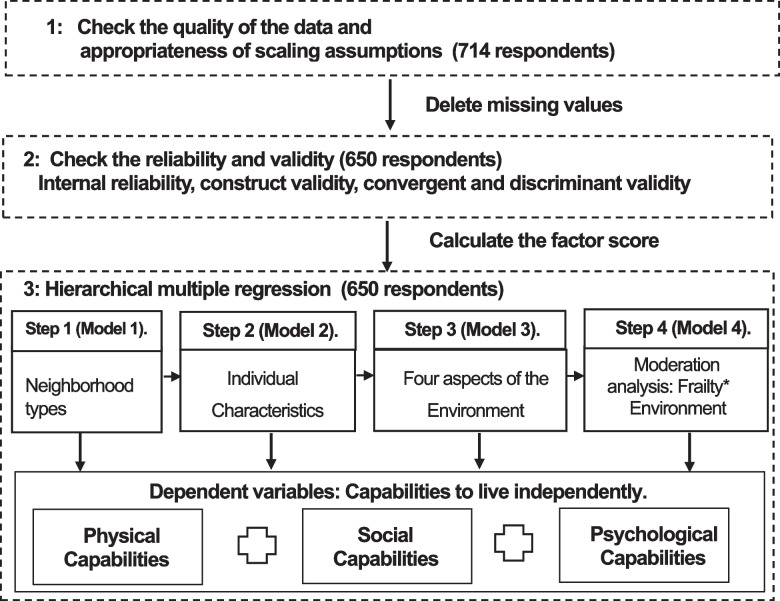
Methodological guideline

Figure 3 presents the methodological steps of the data analysis. First, we checked the data quality and appropriateness of the scaling assumptions by measuring acceptability (completeness and distribution of scores) and precision (how well an item fits within its proposed scale). Acceptability was assessed by calculating the percentage of missing data, floor and ceiling effects (participants' lowest and highest responses, respectively) at the item level (more than 15–20%), and skewness of score distributions (limits: − 1 to + 1). Precision was assessed by calculating item-total correlations [48]. Due to missing values, 64 questionnaires were excluded. The following calculations were based on 650 respondents. The second is the scale validation. We conducted reliability and validity tests to check internal reliability, construct validity, convergent validity, and discriminant validity. Internal reliability refers to the internal consistency of a measurement, which is assessed by Cronbach's alpha (values over 0.8 are acceptable) [49]. Construct validity concerns the extent to which your test or measure accurately what it's supposed to, which was assessed by correlations (0.1–0.6 was an acceptable range) [49]. Convergent validity measures how closely the scale is related to other variables and other measures of the same construct., which was assessed by AVE score (values over 0.5 are acceptable) and composite reliability score (values over 0.7 are acceptable) [49]. Discriminant validity indicates the degree to which a test does not correlate with other tests that measure different constructs, which can be assessed by examining whether the square root of the AVE scores was higher than correlations [49]. After checking the factor structure by using confirmatory factor analysis, we used an aggregate score for each construct, a higher score denotes higher capability levels and a better residential environment. The third step included three hierarchical multiple regression models using physical (model 1a), social (model 1b), and psychological capabilities (model 1c) as dependent variables in R. Each hierarchical regression analysis included neighborhood-type dummies in Model 1 to examine the capability differences between different neighborhood types. Model 2 included individual characteristics. Age was not included since frailty is highly correlated with age. Model 3 included four environmental factors to examine the impact of the residential environment on older people's capabilities to live independently. Model 4 included the interaction terms between frailty and environmental factors to investigate the moderation effects of frailty.

## Results

### Scale validation

The measurement scale was generally acceptable and precise (Table 2). Missing data rates ranged from 0% to 0.42%. Floor effects varied from 0.98% to 13.03%, with the highest percentage occurring among items in the pleasant surroundings subscale. Ceiling effects varied from 7.84% to 25.35%. The only area that did not meet the threshold (15–20%) was the life convenience subscale, where four items failed to meet the criterion. Despite psychometric violation, such a result can be conceptually justified. Beijing's city environment provides reasonable access to the necessary amenities, such as bus stops, grocery stores, pharmacies, and other services, all within walking distance. Score distributions for capability measurement and environmental factors were acceptable (skewness ranges from -0.46 to 0.11). Item-total correlations ranged from 0.57 to 0.79, indicating a good fit.

**Table 2:** Capability and residential environment measurement: acceptability and precision

	**Missing value**	**Mean**	**SD**	**Skewness**	**Floor effect** **%**	**Ceiling effect %**	**Correlated** **Item-total** **Correlation**
Physical capability							
Phy1	0.14	3.28	1.02	-0.09	3.22	11.91	0.76
Phy2	0.28	3.11	1.11	0.02	6.58	11.63	0.67
Phy3	0.14	3.20	1.12	-0.13	7.00	13.17	0.73
Phy4	0.28	3.02	1.09	0.10	6.72	9.66	0.75
Social capability							
Soc1	0.28	3.33	0.94	-0.10	2.10	10.50	0.60
Soc2	0.42	3.09	1.06	0.06	5.60	10.64	0.63
Soc3	0.28	3.00	1.09	0.08	7.28	9.10	0.61
Soc4	0.28	3.17	1.05	-0.13	6.02	10.08	0.62
Psychological capability							
Psy1	0.00	3.56	1.02	-0.35	2.52	18.91	0.66
Psy2	0.14	3.45	1.06	-0.31	3.92	16.95	0.65
Psy3	0.14	3.51	1.05	-0.40	4.06	18.21	0.73
Psy4	0.42	3.46	1.02	-0.29	2.80	15.27	0.71
Perceived Accessibility							
PA1	0.28	3.30	1.10	-0.23	7.00	16.53	0.59
PA2	0.42	3.21	1.11	-0.20	7.14	12.33	0.65
PA3	0.42	3.15	1.11	-0.16	8.54	11.63	0.69
PA4	0.28	3.18	1.18	-0.21	9.38	13.17	0.78
PA5	0.28	3.17	1.16	-0.19	8.96	12.61	0.79
Pleasant surrounding							
PS1	0.28	2.96	1.19	0.05	12.18	11.35	0.71
PS2	0.00	2.92	1.21	0.11	13.03	11.77	0.72
PS3	0.28	2.99	1.05	0.10	6.30	7.84	0.68
PS4	0.14	2.86	1.15	0.10	12.46	8.26	0.76
Meeting opportunities							
MO1	0.00	3.39	1.02	-0.45	5.04	11.91	0.57
MO2	0.14	3.23	1.13	-0.04	6.16	15.97	0.61
MO3	0.14	3.35	1.07	-0.32	5.32	13.73	0.72
MO4	0.14	3.38	1.05	-0.40	5.18	12.89	0.74
Life Convenience							
LC1	0.14	3.76	0.99	-0.45	0.98	25.35	0.70
LC2	0.28	3.62	1.05	-0.38	2.24	22.83	0.66
LC3	0.14	3.64	1.06	-0.46	2.94	23.53	0.69
LC4	0.28	3.76	0.94	-0.46	0.98	22.69	0.76

The full measurement model showed a good model fit (For older people's capabilities to live independently, CFI = 0.991, TLI = 0.989, RMSEA = 0.030, SRMR = 0.031; For environmental factors, CFI = 0.965, TLI = 0.958, RMSEA = 0.051, SRMR = 0.050). In all cases, the factor loadings were above 0.6, indicating that the proposed items are valid indicators of the preconceived theoretical structure. A factor analysis with eigenvalues > 1 indicated a variance explained by the first factor lower than 50%, indicating no common method bias problem [36].

The reliability and validity of the items were confirmed (Table 3). First, Cronbach's alpha coefficients were greater than 0.8 in all cases, indicating that measurable items have good internal reliability [36]. Second, the correlation test fell within an acceptable range (0.1–0.6), indicating that these dimensions measure different theoretical constructs but are correlated. Third, the AVE scores were higher than 0.5, the composite reliability scores were higher than 0.7, and the square root of the AVE scores were higher than correlations between different dimensions [36]. This finding indicated that the measurable items have good convergence and discriminate validity.

**Table 3:** The results of the reliability and validity test

	**Cronbach's alpha**	**Composite Reliability**	**AVE**	
**Capability**	0.875			
Physical capability	0.873	0.875	0.636	
Social capability	0.801	0.801	0.503	
Psychological capability	0.852	0.853	0.592	
**Environmental factors**	0.859			
Perceived accessibility	0.879	0.881	0.602	
Pleasant surroundings	0.868	0.870	0.628	
Meeting opportunities	0.826	0.830	0.555	
Life convenience	0.855	0.855	0.596	
**Discriminant validity score:**				
**Capability**	**Physical capability**	**Social capability**	**Psychological capability**	
Physical capability	0.798			
Social capability	0.455	0.709		
Psychological capability	0.498	0.566	0.770	
**Environmental factors**	**Perceived accessibility**	**Pleasant surroundings**	**Meeting opportunities**	**Life convenience**
Perceived accessibility	0.776			
Pleasant surroundings	0.562	0.792		
Meeting opportunities	0.134	0.168	0.745	
Life convenience	0.188	0.318	0.246	0.772

### Hierarchical multiple regression

#### Capability differences between different neighborhood types

Table 4 presents the results of the hierarchical regression analysis. Model 1 confirmed differences between the neighborhood types with respect to the mean capability level. Such capability differences can only partly be attributed to a neighborhood's socio-demographic composition. In Model 2, although adding individual characteristics resulted in a decrease in the regression coefficients for neighborhood dummies, almost all of them remained significant. The residential environment is dominant in explaining capability differences between neighborhood types. When Model 3 included environmental factors, the effects of neighborhood types on older people's capabilities were no longer significant.

**Table 4:** Results of the hierarchical multiple regression

Dependent Variable	Physical Capability					Social Capability					Psychological Capability				
	Model 1a	Model 2a	Model 3a	Model 4a		Model 1b	Model 2b	Model 3b	Model 4b		Model 1c	Model 2c	Model 3c	Model 4c	
	B	B	B	B	S.E	B	B	B	B	S.E	B	B	B	B	S.E
(Intercept)	-0.463***	-0.171	0.162	0.150	0.147	-0.264***	-0.789***	-0.547***	-0.553***	0.111	-0.318***	-0.503***	-0.170	-0.162	0.139
**Neighborhood type**: (ref = traditional hutong)															
Former Danwei	0.457***	0.281***	0.123	0.126	0.076	0.313***	0.145*	0.071	0.069	0.057	0.411***	0.243**	0.116	0.110	0.072
Commercial housing	0.694***	0.465***	0.160	0.161	0.089	0.366***	0.145*	0.019	0.013	0.067	0.437***	0.200*	-0.008	-0.018	0.084
Urban village	0.432***	0.237*	0.184	0.191*	0.095	0.195*	0.083	0.091	0.093	0.072	0.170	-0.010	0.018	0.016	0.091
**Frailty**		-0.302***	-0.275***	-0.275***	0.020		-0.098***	-0.077***	-0.075***	0.015		-0.149***	-0.120***	-0.118***	0.019
**Male**		0.030	0.038	0.038	0.047		0.000	0.011	0.014	0.036		-0.036	-0.017	-0.014	0.045
**Local hukou**		-0.068	-0.052	-0.058	0.063		0.109*	0.132**	0.129**	0.047		-0.079	-0.052	-0.052	0.060
**Married**		-0.010	-0.011	-0.013	0.047		-0.027	-0.024	-0.030	0.036		0.088	0.085	0.079	0.045
Education:(ref = max. primary school)															
Junior high or senior high		0.082	0.069	0.068	0.056		0.106*	0.085*	0.084*	0.042		0.054	0.030	0.029	0.053
University or above		-0.017	-0.010	-0.014	0.077		0.139*	0.149*	0.149*	0.058		0.094	0.108	0.111	0.073
Income a: (ref = < 3000 yuan)															
3000–5000 yuan		0.056	0.053	0.056	0.068		0.016	0.023	0.017	0.051		-0.029	-0.029	-0.038	0.064
≥ 5000 yuan		0.139	0.068	0.066	0.073		0.082	0.018	0.007	0.055		0.137	0.045	0.033	0.069
**Social Network**		0.019***	0.014***	0.014***	0.004		0.032***	0.023***	0.024***	0.003		0.023***	0.014***	0.014***	0.004
**Instrumental help**		0.046*	0.013	0.015	0.020		0.032*	0.015	0.018	0.015		0.064**	0.032	0.033	0.019
**Perceived accessibility**			0.238***	0.240***	0.046			0.126***	0.135***	0.035			0.195***	0.206***	0.044
**Pleasant surroundings**			0.079*	0.075*	0.037			0.065*	0.058*	0.028			0.066	0.060	0.035
**Meeting opportunities**			0.038	0.038	0.044			0.256***	0.252***	0.033			0.188***	0.185***	0.042
**Life convenience**			0.089*	0.091*	0.038			0.049	0.051	0.029			0.153***	0.155***	0.036
Frailty* Home accessibility				-0.004	0.031				0.000	0.023				0.020	0.029
Frailty * Pleasant surroundings				-0.024	0.027				-0.022	0.020				-0.018	0.025
Frailty * Meeting opportunities				0.021	0.033				0.090***	0.025				0.086**	0.031
Frailty * Life convenience				0.004	0.027				-0.001	0.021				-0.007	0.026
N (Observations)	650	650	650	650		650	650	650	650		650	650	650	650	
**Adjusted R**^**2**^	0.076	0.387	0.452	0.450		0.041	0.293	0.417	0.426		0.050	0.242	0.362	0.366	

Significance levels: * *p* < 0.05, **p < 0.01; ***p < 0.001

#### Determinants of capabilities

Of all of the included variables in Model 2, only three variables, frailty, social networks, and adult children's instrumental help, were significant. The significant effects were all in the expected direction. According to the standardized BETA of Model 3 (not shown), frailty is the strongest predictor of older people's capabilities. As frailty measures physical aspects of health, it is unsurprising that frailty strongly affects physical capability (B = -0.302; *p* < 0.001). Notably, the effects of frailty from a biomedical perspective on social capability (B = -0.098; *p* < 0.001) and psychological capability (B = -0.149; *p* < 0.001) were also significant. The social network had a lower significance level in each of the models than instrumental help, and its effect was even stronger on social capability (B = 0.032; *p* < 0.001) than on psychological capability (B = 0.023; *p* < 0.001) and physical capability (B = 0.019; *p* < 0.001). There was no significant effect on gender, income, or being married or not. Surprisingly, socioeconomic status hardly affected the capabilities of older people. Model 2b only revealed that people with higher education and local hukou (B = 0.109, *p* < 0.05) have higher social capabilities.

The results of Model 3 indicated that four environmental factors positively affect older people's capabilities. The variance accounted for in the models is substantially increased by incorporating these environmental features. Perceived accessibility was the only factor to significantly impact each type of capability (physical, social, and psychological). Meeting opportunities had a greater impact on social and psychological capability (B = 0.256, *p* < 0.001; B = 0.188, *p* < 0.001; respectively). Pleasant surroundings affected physical and social capabilities (although the effects are weaker) but not psychological capabilities. Life convenience (access to facilities) was very important for psychological capability.

#### Moderation analysis

Two significant interaction terms showed up in Model 4: the interaction effect of meeting opportunities and frailty on older people's social capability (B = 0.090, *p* < 0.001) and psychological capability (B = 0.086,

*p* < 0.01). The interaction terms indicate that meeting opportunities have a stronger impact on the social and psychological capabilities of frail older people.

## Discussion

It has been criticized that the traditional health definition has deficiencies because chronic conditions cannot be reversed by medical treatments. We thus refer to Huber's positive health definition, arguing that health is not the absence of disease but the ability to adapt and self-manage in the face of social, physical, and emotional challenges. There is a need to develop a community-based approach to healthy aging that enables older people to adapt and self-manage. Drawing on Sen's capability approach, we propose that such a community-based approach should provide a supportive environment to enable older people's capabilities to live independently. Using hierarchical multiple regression analysis, this study discussed the differential impacts of four environmental factors (perceived accessibility, pleasant surroundings, meeting opportunities, and life convenience) that enable older people's capabilities to live independently. We also examined how frailty affects the degree to which the residential environment affects older people's capabilities.

The results show that four environmental factors positively affect Chinese older people's capabilities to live independently. Despite studies suggesting that supportive residential environments can influence older people's health and well-being [6, 7, 42], according to Sen's capability approach, we argue that policy should focus on older people's capabilities rather than their valued functionings. It should be noted that perceived accessibility was the only environmental factor to have a significant impact on each type (physical, social, and psychological) of capability. Although previous research indicates that home modifications are essential in supporting older people [27, 35, 37], this study further shows that perceived accessibility enhances aging in place by enabling older people's capabilities to live independently. Apart from perceived accessibility, this study highlights the role of meeting opportunities in supporting older people's capabilities to live independently. Previous studies have discussed the crucial role that the social environment plays in supporting healthy aging [28, 29, 42]. It has not been fully explored how this social infrastructure assists older people in achieving their valued functionings [14]. This study found that meeting opportunities do not affect the physical capability of older people, but they significantly contribute to older people's social and psychological capabilities.

Four environmental factors we identified explain capability differences between different neighborhood types. In the literature, health policy for older people focuses on individual behaviors, whereas less attention is paid to structural and material barriers [50]. Our findings however, support the argument that inequalities in health may be usefully explained by inequalities in capabilities, and that differences in older people’s capabilities are highly related to both physical and social aspects of the residential environment. More specifically, the results show that the capability differences between different neighborhood types can only be partially explained by the socio-demographic composition of the neighborhood. The residential environment is dominant in explaining capability differences between neighborhood types. In other words, we should focus on contextual approaches that emphasize the influence of environment on older people’s capabilities rather than compositional approaches that emphasize the influence of the individual's personal characteristics on his/her capabilities.

The capability approach has the advantage of highlighting human diversity. Such an advantage is crucial because it addresses a main criticism of mainstream frameworks: their inability to appreciate and incorporate diversity when examining experiences of aging [12]. This study found no interaction effect for perceived accessibility, pleasant surroundings, and life convenience, implying that these environmental conversion factors are equally important for frail and non-frail older people. That is to say, physical health decline is not the only problem associated with aging. Even non-frail older people need environmental support. Nevertheless, in line with the literature that has already shown that older people's psychological and social needs vary greatly based on their frailty level [17], the results of moderation analysis show that meeting opportunities significantly impact frail older people's psychological and social capabilities. Frail older people may cut out peripheral social relationships and show a greater focus on access to maintaining nearby relationships [51]. Therefore, frail older people are more susceptible to the impact of meeting opportunities.

This study has policy implications as Chinese policymakers are exploring effective community-based environmental support for older people [28, 39, 45]. The finding of this study suggests that the policy focus is to support older people's capabilities to live independently. The government is advised to consider the importance of the usability of a home, the landscape design of residential areas, the creation of meeting opportunities, and the establishment of urban infrastructure for life convenience. Since different neighborhood types have different capabilities, community-based policy interventions need to consider contextual appropriateness. In particular, urban renewal programs are supposed to focus primarily on improving conditions in traditional neighborhoods. It is because results show that respondents living in traditional neighborhoods scored lower in capabilities than those living in former danwei compounds and commercial housing. Considering frail older people are more sensitive to meeting opportunities, the Chinese government is responsible for collecting opinions and considering the social needs of frail older people.

This study has limitations because the required capability set can vary depending on the cultural context. This paper developed a capability measurement and evaluated its psychometric properties. This measurement was psychometrically robust, meeting reliability, validity, and precision criteria. It may be hypothesized that, however, Chinese older people attach more importance to caring about society and making a meaningful contribution. It would be interesting to investigate how older people in different cultural contexts value different forms of capabilities that enable them to live independently.

## Conclusion

The study contributes to the literature by suggesting a community-based approach that provides a supportive environment to enable older people to adapt and self-manage. Drawing on the capability approach, this study argues that instead of focusing on the valued functionings of older people, we should focus on their capabilities to live independently. We developed a capability measurement and evaluated its psychometric properties. This measurement was psychometrically robust, fully meeting reliability, validity, and precision criteria. Our focus is on the importance of the residential environment in supporting older people's capabilities rather than individual behavior or choices. The findings show that the residential environment, not the socio-demographic composition of the neighborhood, plays the most significant role in explaining the capability differences between different types of neighborhoods. We identify four aspects of environmental factors (perceived accessibility, pleasant surroundings, meeting opportunities, and life convenience) that positively affect Chinese older peoples capabilities to live independently. The study also discusses how the residential environment can affect a person’s capabilities to live independently depending on their level of frailty. We pointed out that frail older people are more susceptible to the impact of meeting opportunities. Results can be used to develop more effective community-based environmental support to enable older people to live independently.

## Data Availability

The datasets generated and/or analyzed during the current study are available from the. the corresponding author on reasonable request.
